# A Review of Neoadjuvant Therapy for Localized and Locally Advanced Renal Cell Carcinoma

**DOI:** 10.3390/cancers17020312

**Published:** 2025-01-19

**Authors:** Qian Qin, Isamu Tachibana, Vitaly Margulis, Jeffrey A. Cadeddu, Tian Zhang

**Affiliations:** 1 Division of Hematology and Medical Oncology, Department of Internal Medicine, University of Texas Southwestern, Dallas, TX 75235, USA; qian.qin@utsouthwestern.edu; 2 Department of Urology, University of Texas Southwestern, Dallas, TX 75235, USA; isamu.tachibana@utsouthwestern.edu (I.T.); vitaly.margulis@utsouthwestern.edu (V.M.); jeffrey.cadeddu@utsouthwestern.edu (J.A.C.)

**Keywords:** pre-operative, neoadjuvant, vascular endothelial growth factor receptor-tyrosine kinase inhibitor, immune checkpoint inhibitor, renal cell carcinoma, kidney cancer

## Abstract

The introduction of vascular endothelial growth factor receptor-tyrosine kinases (VEGFR-TKIs) and immune checkpoint inhibitors (IOs) have drastically altered the treatment landscape for kidney cancer, with doublet combination immunotherapy (IO/IO or IO/VEGFR-TKI) now set as the standard front-line treatment for advanced renal cell carcinoma (RCC). However, the roles of VEGFR-TKIs and IOs in the neoadjuvant setting for locoregional/locally advanced RCC remain undefined, where the goals may be primary tumor downsizing/downstaging and potentially eradicating micrometastatic disease. This review will examine VEGFR-TKI monotherapy, IO monotherapy, and VEGFR-TKI/IO combination regimens in a preoperative setting with a focus on the efficacy, toxicity, surgical, and long-term implications.

## Simple Summary

With the discovery of immunotherapy (treatments that increase the immune system’s ability to kill cancer cells) and targeted therapy (treatments that target the specific pathway the cancer uses to grow and survive), we have made great progress in the treatment of advanced kidney cancer. However, their roles in a localized setting (a tumor limited to the kidney and/or lymph nodes around the kidney) are not well defined. This review will look at the available data on the use of immunotherapy and targeted therapy for patients with localized kidney cancer, where the goals may be to decrease tumor size, to make surgery easier and less invasive, to save as much kidney function as possible, and/or to kill the cancer cells that may have escaped the kidney.

## 1. Introduction

**Figure 1:**
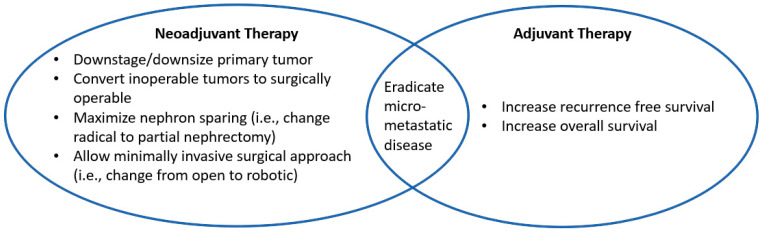
Goals of neoadjuvant and adjuvant therapy for renal cell carcinoma.

In the United States, an estimated 81,610 patients will be diagnosed and 14,390 will die from kidney cancer in 2024 [1]. Kidney cancer is often detected in the locoregional stage, with approximately 66% in the localized (confined to the primary site) and 16% in the regional (spread to the regional lymph nodes) stages, while approximately 15% are de novo metastatic [1]. Doublet combination immunotherapy (immune checkpoint inhibitor [IO]/IO and IO/vascular endothelial growth factor receptor-tyrosine kinase inhibitor [VEGFR-TKI]) is now the standard front-line treatment for advanced renal cell carcinoma (RCC). For subsequent therapies, additional options include everolimus (an inhibitor of the mammalian target of rapamycin) and belzutifan (an inhibitor of hypoxia-inducible factor-2 alpha [HIF2α]), given as a monotherapy treatment or in combination regimens. However, the roles of VEGFR-TKI, IO, and novel agents for locoregional/locally advanced diseases in a perioperative setting are still being defined (Figure 1). Although KEYNOTE-564 led to the approval of pembrolizumab in adjuvant setting, its clinical use may not be widespread, even with the updated OS data, in part due to several other negative and ongoing adjuvant/perioperative trials [2,3,4,5,6,7,8].

Furthermore, significant questions remain regarding the role of neoadjuvant therapy, where the goals may be tumor downsizing/downstaging and potentially eradicating micrometastatic disease. Tumor downsizing/downstaging may allow minimally invasive and/or nephron-sparing approaches, though the safety and potential complications introduced to the subsequent surgery need to be evaluated. This review will examine the utility of VEGFR-TKI and IO therapies in this preoperative space with a focus on patients with localized and locally advanced RCC.

## 2. TKI Monotherapy

The vascular endothelial growth factor (VEGF) belongs to the VEGF/platelet-derived growth factor (PDGF) family of the cystine-knot superfamily. They are important signal proteins in the vasculogenesis and angiogenesis pathways, where their dysregulation are key drivers of RCC growth and progression. In addition to the anti-VEGF monoclonal antibody, bevacizumab, the development of VEGFR-TKIs has expanded therapeutic options for advanced RCC. Their utility in the perioperative space is under evaluation. Though no phase III trials have been conducted thus far, pilot and phase II trials have been implemented to test the efficacy, safety, and feasibility of neoadjuvant VEGFR-TKI monotherapies (Table 1). Particularly, the ability of VEGFR-TKI to downsize/downstage the primary tumor with potential to maximize renal function sparing is an endpoint of high interest. However, potential surgical delay and wound healing complications are of concern given inhibition of the angiogenesis pathway.

### 2.1. Sorafenib

One of the initial VEGFR-TKIs utilized in advanced RCC, sorafenib inhibits intracellular Raf kinases (CRAF, BRAF, and mutant BRAF) and cell surface kinase receptors (VEGFR-1, VEGFR-2, VEGFR-3, PDGFR-beta, cKIT, FLT-3, RET, and RET/PTC) [21]. The effect of sorafenib in the neoadjuvant space was evaluated in two pilot trials. In the first open-label pilot trial, Cowey et al. treated 30 patients with at least stage II RCC (n = 17 localized and n = 13 metastatic) with sorafenib 400 mg orally (PO) twice a day (BID) until their planned nephrectomy [9]. After a median time on sorafenib of 33 days (range 8–59 days), a majority of the patients (83%) achieved primary tumor shrinkage with median reduction of −9.6% (range −40% to +16%) and 7% (2/30) achieving a partial response (PR) per the Response evaluation Criteria in Solid Tumors (RECISTs). The most common adverse events (AEs) included fatigue, nausea, diarrhea, rash, stomatitis, hypertension, and hand–foot syndrome with no grade 4/5 toxicities attributed to sorafenib. All patients underwent radical nephrectomy (RN, 53% laparoscopic and 47% open) with 33% requiring a caval thrombectomy and 17% undergoing adrenalectomy. No significant complications such as delayed wound healing, surgical dehiscence, or excessive bleeding were observed [9].

A subsequent study by Hatiboglu et al. showed higher response to sorafenib in a placebo-controlled, double blind, pilot trial randomizing patients with localized RCC (cT1-3, N0, M0) to 4 weeks of sorafenib (400 mg PO BID) versus placebo (3:1 randomization) [10]. For the nine patients receiving sorafenib, the median primary tumor size reduction was −29% (−61.1% to −4.9%), with 44% (4/9) achieving PR. No tumor size reduction was observed in the placebo group (range 0 to +24.2%). AEs were consistent with the safety profile of sorafenib. No surgical complications, unusual bleeding, or delay in wound healing were observed [10].

As one of the first VEGFR-TKIs to be utilized in the neoadjuvant space, sorafenib demonstrated preliminary efficacy and feasibility of utilizing VEGFR-TKI in the neoadjuvant setting.

### 2.2. Sunitinib

Sunitinib is a VEGFR-TKI with activity against platelet-derived growth factor receptor (PDGFR)-α and -β, VEGF receptors (VEGFRs), stem cell factor receptor (c-KIT), FMS-like tyrosine kinase 3 (FLT3), colony-stimulating factor-1 receptor (CSF-1R), and Ret Proto-Oncogene (RET) [22]. Sunitinib is approved for the treatment of RCC in adjuvant and advanced settings, though its clinical utilization has decreased as newer VEGFR-TKIs have become available and immunotherapy doublets have shown better efficacy over sunitinib in controlled cohorts. Several small studies have evaluated sunitinib in the neoadjuvant setting with varying response rates.

Two small, pilot trials conducted by Silberstein et al. (n = 12) and Hellenthal et al. (n = 20) evaluated sunitinib 50 mg 4 weeks on/2 weeks off (4w:2w) for two cycles and sunitinib 37.5 mg PO daily for three months, respectively, in patients with localized or metastatic clear cell RCC [11,12]. In Silberstein et al., 28.5% of the patients achieved PR, and the mean primary tumor reduction was −21.1% (range −45% to −3.2%) across all patients [11]. Response was more modest in Hellenthal et al., where only 5% achieved PR and the median primary tumor reduction was −11.8% (−27% to +11%), though eight patients with tumor size reduction were able to undergo laparoscopic PN and no major surgical complications were observed [12].

Subsequently, Rini et al. conducted a larger, phase II trial (NCT00459979) that enrolled 30 patients with locally advanced or metastatic RCC with any histology [13]. Patients received sunitinib 50 mg 4w:2w (first n=9 patients), followed by an increase in dose to 50 mg daily due to tumor progression noted during the 2 weeks off, until surgery, unacceptable toxicity, or disease progression [13]. In the 28 evaluable patients, the median reduction in primary tumors was −22% (range −100% to +13%), with 7/28 patients (25%) achieving PR [13]. The clear cell histology tumors had a greater tumor size reduction (median −28%) when compared to the non-clear cell histology tumors (median +1.4%). In the 13 patients who underwent surgical resection (n = 4 RN and n = 9 PN), the median primary tumor change was −27% (versus −11% in those who did not undergo surgery). Toxicity was consistent with known AEs of sunitinib, and no major wound healing complications or thromboembolic events occurred [13].

The varying responses to sunitinib observed are unsurprising given the small sample sizes, varying disease stages/subtypes enrolled, as well as varying dose/time on therapy. However, downsizing/downstaging was seen in all of the trials, and a portion underwent PNs, indicating the potential debulking roles of neoadjuvant VEGFR-TKI.

### 2.3. Other VEGFR-TKIs

Following the preliminary efficacy seen with sunitinib, Rini et al., Karam et al., Lebacle et al., and Bilen et al. specifically evaluated the ability of VEGFR-TKIs to downsize/downstage localized, clear cell RCC and, potentially, maximize renal function preservation [14,15,16].

In a phase II study, Rini et al. treated localized RCC patients with 8 to 16 weeks of pazopanib 800 mg PO daily [14]. A total of 25 patients were enrolled (n = 24 clear cell RCC and n = 1 chromophobe RCC), of whom 56% had solitary kidney and 56% had preexisting chronic kidney disease. A majority of the tumors (all except two) were downsized, with a median reduction in tumor diameter of −26% and a PR rate of 36% [14]. Of the 13 patients enrolled who required RN at baseline, 6 (46%) were successfully downsized to undergo a partial nephrectomy (PN) [14]. Furthermore, the amount of functional parenchyma saved by PN, assessed by a volumetric computed tomography (CT) scan, increased by 62% (from mean of 107 cc at baseline to 173 cc after treatment with pazopanib, *p* = 0.0015) [14]. Only one patient who received PN required dialysis; the other patients had sufficient renal preservation to preclude renal replacement therapy. Grade 3 AEs occurred in 64% of the patients, most frequently hypertension (36%) and elevated liver enzymes (20%). No grade 4/5 AEs were observed. Perioperative events such as transfusion (n = 7) and urine leaks (n = 5) were potentially higher compared to the historical control of PN without neoadjuvant VEGFR-TKI, though no Clavien grade 4/5 perioperative AEs, thromboembolic events, or long term sequala occurred [14].

Karam et al. (n = 24, NCT01263769) and Lebacle et al. (n = 18, NCT02597322) evaluated the efficacy of axitinib 5 mg PO BID (dose up titration allowed) for 12 weeks and up to 6 months, respectively, in patients with localized, clear cell RCC [15,16]. In the Karam et al. trial, all tumors showed shrinkage with a median primary tumor reduction of −28.3% (range −42.9% to −5.3%) and a PR rate of 45.8% [15]. All enrolled patients had clinical T3a disease and, at surgical resection, 21% (n = 5) of the patients were noted to have pathologic T1-2 disease and 21% (n = 5) underwent PN. AEs were consistent with the known toxicities of axitinib, and no delay in surgery or intraoperative complications were noted. Notable postoperative complications included chylous ascites (n = 3), bleeding (n = 1), pulmonary embolism (n = 2), and superficial wound dehiscence (n = 1), all of which were managed without long term sequala [15]. Subsequently, Lebacle et al. evaluated up to 6 months of axitinib in 18 patients with localized, clinical T2a clear cell RCC and considered not suitable for PN [16]. A majority of the tumors decreased in size with a median reduction of −17.1% (range −29.4 to +4.8) and an objective response rate (ORR, all PR) of 22%. Sixty-seven percent (12/18) of the patients achieved the primary outcome of PN for a tumor < 7 cm in size. A majority of the axitinib treatment-related AEs were grade 1/2 with the most common being hypertension, fatigue, dysphonia, and hand–foot syndrome. No grade 4/5 events and no wound or abdominal wall complications were seen. Postoperatively, 11 patients experienced Clavien grade I/II complications and 5 experienced Clavien grade III–V complications (embolization for severe bleed, urine leakage, suicide attempt, and death due to myocardial infarction) [16].

Most recently, cabozantinib was evaluated in a phase II, neoadjuvant trial (NCT04022343) that enrolled patients with locally advanced (clinical stage ≥ T3Nx or TanyN+ or deemed unresectable), nonmetastatic clear cell RCC [17,18]. Seventeen patients were enrolled and treated with cabozantinib 60 mg PO daily for 12 weeks, followed by a 4-week wash-out and surgical resection. All patients had primary renal tumor size reduction with a median of −26% (range −42 to −8%) and an ORR of 35% (all PR) [18]. One patient with unresectable tumor at enrollment became resectable after treatment, and two patients converted from RN intent to PN. Most common AEs included diarrhea, nausea, fatigue, hypertension, anorexia, and hand–foot syndrome. No cabozantinib-associated grade 4/5 treatment related AEs and no perioperative or postoperative surgical complications were observed. The investigators also evaluated long term outcomes including one-year disease-free survival (DFS, 82.4%) and one-year overall survival (OS, 94.1%). Interestingly, the investigators also noted a trend towards CD8+ T cell activation post-cabozantinib therapy.

Overall, the above studies demonstrated the feasibility and preliminary efficacy of neoadjuvant VEGFR-TKI monotherapies, with a majority of the tumors showing shrinkage and some achieving PR, though they rarely led to complete responses (CRs). Importantly, the trials showed the potential role of VEGFR-TKI in reducing tumor burden and minimizing surgical field/maximizing renal parenchyma preservation. Given the higher efficacy seen in clear cell when compared to non-clear cell subtypes, later trials focused their enrollment on clear cell RCC with potentially higher response rates. However, small sample sizes and significant variability in setting (localized, locally advanced, with or without metastatic disease)/duration of therapy (weeks to months) preclude more definitive conclusions. Furthermore, variable perioperative and postoperative complications were reported, but whether and which of these may correlate with VEGFR-TKI use is unclear. AEs were consistent with known toxicities of VEGFR-TKI, which tend to recover after discontinuation. Unmet needs include correlative studies of intra-tumoral and tumor microenvironment changes with VEGFR-TKI and larger contemporary trials in combination with immunotherapy.

## 3. IO Single Agent and Combination Therapy

The development of IO has drastically changed the landscape of RCC treatment in recent years, with IO/IO (ipilimumab/nivolumab) approved in the front line setting for advanced clear cell RCC. IO is also being extensively studied in the adjuvant space for high-risk localized RCC, with adjuvant pembrolizumab approved based on DSF and OS benefits seen in the phase III KEYNOTE-564 study [2,3]. However, this is in the context of other negative or ongoing trials [4,5,6,7,8].

The effect of IO in the neoadjuvant setting is less explored, limited to two single arm, pilot trials evaluating the use of nivolumab in patients with localized RCC (Table 1) [19,20]. In Gorin et al., 17 patients with nonmetastatic high-risk RCC (T2a-T4NanyM0 or TanyN1M0) were enrolled and treated with nivolumab 3 mg/kg intravenous (IV) every 2 weeks for three cycles followed by surgical resection (NCT02575222) [19]. In Carlo et al., 18 patients with localized, clear cell RCC and at high risk of recurrence (≥20% risk as estimated by a preoperative nomogram) were enrolled and treated with nivolumab IV every 2 weeks for four cycles followed by surgical resection (NCT02595918) [20,23]. Both trials showed the feasibility of neoadjuvant nivolumab, with all patients receiving three doses of nivolumab in Gorin et al. and 94% receiving at least three doses in Carlo et al. [19,20]. However, minimal efficacy was observed, with all evaluable patients having stable disease (SD) without objective responses (Table 1) [19,20]. Immune-related AEs (irAEs) were observed and consistent with known toxicity of nivolumab. In Gorin et al., though no grade 4/5 events occurred, 82.4% of patients experienced AEs, with fatigue, pruritis, and rash being the most common [19]. No major perioperative complications occurred, and no patient experienced a Clavien grade ≥ 3 complication [19]. In Carlo et al., two patients required systemic corticosteroids for irAEs (grade 3 transaminitis and grade 2 intolerable arthralgias) and two developed delayed irAEs (grade 3 colitis and acute kidney injury) [20]. No significant increase in intraoperative and postoperative complications were seen, with one patient receiving intraoperative blood transfusion and two patients who underwent lymph node dissections developing chylous leak [20]. On long term follow up, Gorin et al. estimated a 3-year metastasis-free survival rate of 85.1% and an OS of 85.7%, while Carlo et al. noted a 1-year recurrence-free survival rate of 82% [19,20]. These trials showed the feasibility of nivolumab monotherapy in the neoadjuvant setting, though with a minimal primary tumor response and not insignificant AEs.

Given the DFS and OS benefits seen with pembrolizumab in the adjuvant setting, one may infer a continuum of IO in the perioperative setting, particularly with a neoadjuvant addition where the primary tumor is intact, could better prime the immune response and improve long-term efficacies (Figure 1). An early phase, multi-cohort trial (NCT02762006) evaluated durvalumab +/− tremelimumab for one dose in the neoadjuvant setting followed by one dose to one year in the adjuvant setting [24,25,26]. The trial showed the feasibility of combination IO without significant surgical delays or complications, though due to higher-than-expected irAEs the study was suspended after accruing 29 patients [24,25,26]. On correlative analysis, the investigators noted immune checkpoint molecule changes (programmed death-ligand 1 [PD-L1] and V-domain Ig suppressor of T cell activation [VISTA]) in the myeloid-derived suppressor cells, particularly a decrease in the frequencies of PD-L1 expression in the peripheral blood post-neoadjuvant IO therapy [24]. The potential long-term effects of these changes remain to be defined.

Subsequently, the phase III PROSPER ECOG-ACRIN EA8143 trial (NCT03055013) randomized 819 patients with high-risk, localized (≥T2 or T(any)N+) RCC (clear cell or non-clear cell) to surgery versus surgery with one neoadjuvant cycle followed by nine adjuvant cycles of nivolumab at 480 mg IV every 4 weeks [5]. The investigators found no difference between the two groups in terms of recurrence-free survival (hazard ratio [HR] 0.94, 95% confidence interval [CI], 0.74–1.21, and one-sided *p* = 0.32) [5]. The HR for OS was 1.28 (95% CI, 0.84–1.95, two-sided *p* = 0.26) and the median OS was not reached in either group. AEs were consistent with known toxicity of nivolumab with 48% of the patients in the nivolumab plus surgery arm experiencing Grade 3-5 irAE versus 24% in the surgery only group, with anemia, hypertension, and elevated lipase being the most common.

Other immunotherapy-based combination trials are ongoing, including the NESCIO trial (NCT05148546), evaluating neoadjuvant nivolumab with or without ipilimumab or relatlimab; the SPARC-1 trial (NCT04028245), evaluating neoadjuvant canakinumab (interleukin-1 beta antagonist) plus spartalizumab (Programmed Cell Death Protein 1 [PD-1] inhibitor); and the NAPSTER trial (NCT05024318), evaluating neoadjuvant stereotactic ablative radiotherapy with or without pembrolizumab (Table 2) [27,28,29,30,31,32]. Long-term follow ups of completed trials and read outs from ongoing trials may help define the role and long-term efficacy of IO therapy in the neoadjuvant/perioperative space.

## 4. VEGFR-TKI/Immunotherapy Combination

Despite the overall negative neoadjuvant IO trials, the promising efficacy of VEGFR-TKI monotherapy and the possible long-term effects of IO therapy led to an increased interest in VEGFR-TKI/IO combination regimens.

The single arm, phase II, NeoAvAx trial (NCT03341845) evaluated 12 weeks of neoadjuvant avelumab (10 mg/kg intravenous every 2 weeks) plus axitinib (5–10 mg PO BID) in 40 patients with high-risk, localized, ccRCC [41]. High risk was defined as cT1b-T2aN0M0 with Fuhrman grade 4, cT2b-T3aN0M0 with Fuhrman grade 3–4, cT3b-T4N0M0 with Fuhrman of any grade, and cTanyN1M0 with Fuhrman of any grade. Forty patients were enrolled with a baseline tumor size of 10.3 cm (range 5.6 to 18.8 cm) and 42.5% had clinical lymph node positive disease. The median primary tumor downsizing was −20% (−43.5% to +3.8%) with 30% (12/40) of the patients achieving PR. At a median follow up of 23.5 months, 67.5% of the patients were disease free overall with higher percentage seen in the patients who achieved PR (83% remained disease free). AEs were consistent with the known toxicities of avelumab and axitinib, with one patient experiencing a 3-week surgical delay due to grade 2 hypothyroidism. Two patients experienced intraoperative AEs (bowel damage and a splenectomy), and five patients experienced Clavien grade ≥ 3 postoperative complications. The investigators also evaluated immune correlates and noted the upregulation of PD-L1 expression (*p* < 0.0001) and total CD8+ densities (*p* < 0.01) in the post-treatment surgical samples when compared to the pre-treatment biopsies. Furthermore, surgical samples of patients who experienced recurrence had lower total/intra-epithelial and stromal CD8+ (*p* < 0.05) and intra-epithelial CD8+CD39+ (*p* < 0.05) densities when compared to patients without recurrence, suggesting possible difference in the expansion of pre-existing immune responses. Overall, the NeoAvAx trial showed the feasibility and promising efficacy of VEGFR-TKI/IO combination in the neoadjuvant setting. AE profile was tolerable but not insignificant, and whether neoadjuvant VEGFR-TKI/IO contributed to intraoperative and perioperative complications is unclear. Long term follow up is also needed to understand the potential effects on DFS and OS.

Additional trials evaluating novel VEGFR-TKI/IO combinations have been completed with varying response rates. A single arm, phase II study (NCT03680521) evaluated up to 8 weeks of neoadjuvant sitravatinib (VEGFR-TKI, 80 mg or 120 mg PO daily) plus nivolumab (240 mg IV every 2 weeks) in 20 patients with locally advanced clear cell RCC [42]. The ORR was modest at 11.8% with a median primary tumor shrinkage of −13.5% (range −33% to 0%) and an estimated 24-month DFS probability of 88% [42]. The combination was overall tolerable without grade 4/5 treatment-related AEs, though four patients had surgical delays, with one patient delaying surgery by 38 days due to nivolumab-related thyroiditis.

A phase II study (NCT04118855) by Huang et al. evaluated up to 12 weeks of toripalimab (PD-1 inhibitor, 240 mg IV every 3 weeks for three cycles) plus axitinib (5 mg PO BID up to 12 weeks) in 20 patients with locally advanced (cT2-T3N0-1M0), clear cell RCC [43]. The median primary tumor reduction was −26.7% (−40.3% to −2%) with an ORR of 45% and four patients achieving pathologic CR at surgical resection [43]. The combination was overall well tolerated with no grade 4/5 AEs; however, one patient had clinical decline and did not undergo surgery, and one patient had delay of surgery due to grade 3 hyperglycemia. At the median follow up of 21.3 months, four patients (20%) experienced recurrence with a median DFS not yet reached. When compared to patients with SD, patients with PR had higher densities of PD-1+, PD-L1+, PD-1+CD8+ cells and M1 macrophages in the tumor tissue obtained prior to treatment [43]. Another phase II study (NCT05172440) by Zhang et al. evaluated 12 weeks of tislelizumab (PD-1 inhibitor, 200 mg IV every 3 weeks for four cycles) plus axitinib (5 mg PO BID) in 20 patients with high-risk nonmetastatic clear cell RCC (cT2a-4 and/or N1, M0) [39]. In the interim analysis with nine patients evaluable for efficacy, the ORR was significant at 55.5% with a median primary renal tumor reduction of −26.3% (range −45% to −12.5%) [39]. Additionally, one patient converted from unresectable to resectable and two patients converting from RN to PN [39]. Most common AEs were hypothyroidism, nausea, vomiting, a decreased appetite, fatigue, diarrhea, elevated ALT/AST levels, and hematologic toxicities with no grade 4/5 AEs and no drug-related surgical complications [39].

Overall, these novel VEGFR-TKI/IO combinations show promising tumor reduction rates. However, longer follow ups and read outs from other, ongoing VEGFR-TKI/immunotherapy combination trials (Table 2) are needed to define their role in the neoadjuvant setting [33,34,35,36,37,38,40].

## 5. IVC Thrombus

Inferior vena cava (IVC) tumor thrombus (TT) occurs in approximately 4–10% of kidney cancer cases and poses unique surgical challenges [44]. Resection requires multi-disciplinary expertise and coordination including but not limited to anesthesia, cardiovascular-thoracic surgery, and urologic oncology. Additionally, patients with high level IVC TT have significantly higher recurrence rates and shorter overall survival [45,46]. Such patients may particularly benefit from neoadjuvant therapy to downstage both the primary tumor and the IVC TT. Retrospective studies have supported this notion, including the study by Tanaka et al. evaluating 41 RCC patients with IVC TT who underwent upfront RN (n = 31) or neoadjuvant axitinib followed by RN (n = 10) [47]. Neoadjuvant axitinib led to a median 21 mm and 54% reduction in IVC TT length and volume, respectively [47]. Furthermore, the neoadjuvant axitinib group had lower blood loss and shorter operative duration when compared to the upfront RN group [47]. However, other small series of neoadjuvant VEGFR-TKI +/− IO reported modest benefit in terms of IVC TT regression and/or changes in surgical approaches [48,49,50]. Prospective trials evaluating neoadjuvant VEGFR-TKI/IO therapies in RCC patients with IVC TT are ongoing (Table 2) [35,37,38]. These include the phase II NEOPAX trial evaluating 12 weeks of pembrolizumab 200 mg IV every 3 weeks plus axitinib 5 mg PO BID and the phase II perioperative trial evaluating 12 weeks of pembrolizumab 200 mg IV every 3 weeks plus lenvatinib 20 mg PO daily followed by adjuvant pembrolizumab [35,37,38]. Results from these prospective studies may shed additional light on the role of neoadjuvant therapy in RCC patients with IVC TT.

## 6. Conclusions

The primary goals of neoadjuvant, systemic therapy include downstaging/downsizing the primary tumor and potentially eradicating micrometastatic disease, thereby improving long-term disease control and survival rates. Though neoadjuvant therapy is the standard of care for many solid tumor types, its role in localized/locally advanced RCC remains to be defined.

Overall, VEGFR-TKIs, with or without IO, showed promising efficacy in downstaging/downsizing primary kidney tumors, with some achieving PR though few achieved CR per RECIST or pathologic CR at surgical resection. In general, although immunogenic, large complex renal masses will not achieve radiographic CR; the ability to downstage/downsize did allow some RN to be converted to PN, with a preservation of renal function. As is shown by Rini et al., this is particularly important in patients with low renal function reserves, where maximum nephron sparing may allow enough renal preservation to avoid renal replacement therapy [14]. Furthermore, the ability of neoadjuvant therapy to convert surgically unresectable (or surgically challenging) patients to resectable is also highlighted by the VEGFR-TKI +/− IO trials. There is a particularly high interest in locally advanced RCC patients with IVC TT, where several trials evaluating the efficacy and surgical implications of VEGFR-TKI/IO combination are ongoing [35,37,38]. However, AEs are not insignificant, and with the anti-angiogenesis effects of VEGFR-TKIs, intraoperative and postoperative complications are of concern. The single-cohort, small sample size nature of reported trials make drawing correlations a challenge, and larger, randomized trials are needed to differentiate the perioperative effects of VEGFR-TKI.

While IO monotherapy has not shown efficacy in primary tumor reduction, IO combined with VEGFR-TKIs may have benefits given the disease control observed in the primary tumors. In particular, the tumor microenvironments with immune-infiltrated tumors tend to have more responses, and IO-TKI combinations have surpassed sunitinib alone in the metastatic setting for early disease control. When early micrometastatic disease can be eradicated, long-term efficacy endpoints such as DFS and OS may also be improved. Ongoing perioperative trials will add to the known improvements in local disease control and inform future trials with longer-term endpoints.

Larger perioperative trials using effective treatments without adding to surgical complications are absolutely needed. Long-term goals of improving DFS and OS remain future possibilities as the field works toward increasing cures while preserving renal function and quality of life of patients.

## Figures and Tables

**Table 1:** Selective neoadjuvant VEGFR-TKI monotherapy and IO monotherapy trials, inclusive of patients with localized kidney cancer.

Trial ^a^	Phase	N	Inclusion Criteria	Localized (M0)	Clear Cell Histology	Agent (Time on tx)	Tx Withdrawal Period Before Surgery	ORR	Reduction of Primary Tumor, Median (Range)
VEGFR-TKI Monotherapy Trials									
Cowey et al. (2010) [9]	Pilot	30	≥cT2, N(any), M(any)	57%	70%	Sorafenib 400 mg BID (until surgery)	24–48 h	7%	−9.6% (−40% to +16%)
Hatiboglu et al. (2017) [10]	Pilot	12 ^b^	cT1-3, N0, M0	100%	83%	Sorafenib 400 mg BID (4 w)	12 h	44% ^b^	−29% (−61.1% to −4.9%) ^b^
Silberstein et al. (2010) [11]	Pilot	12	Localized or metastatic	58%	100%	Sunitinib 50 mg 4 w on/2 w off (2 cycles)	2 w	28.5%	−21.1% (−45% to −3.2%) ^c^
Hellenthal et al. (2010) [12]	Pilot	20	cT1 b-T3, N(any), M(any)	80%	100%	Sunitinib 37.5 mg daily (3 m)	5 d (first n = 5) D prior (n = 15)	5%	−11.8% (−27% to +11%) ^c^
Rini et al. (2012) [13]	II	28	cT(any), N(any), M(any)	34%	76%	^d^ Sunitinib 50 mg 4 w on/2 w off (first n = 9) Sunitinib 50 mg daily (remaining n = 19)	7 d	25%	−22% (−100% to +13%)
Rini et al. (2015) [14]	II	25	^e^ Localized	100%	96%	Pazopanib 800 mg daily (up to 16 w)	7 d	36%	−26%
Karam et al. (2014) [15]	II	24	cT2-3 b, N0, M0	100%	100%	Axitinib 5 mg BID, up titration allowed (12 w)	36 h	45.8%	−28.3% (−42.9% to −5.3%)
Lebacle et al. (2019) [16]	II	18	cT2a, N0-x, M0	100%	100%	Axitinib 5 mg BID, up titration allowed (2, 4, or 6 m)	-	22%	−17.1% (−29.4% to +4.8%)
Bilen et al. (2022) [17,18]	II	17	≥cT3Nx or T(any)N1M0	100%	100%	Cabozantinib 60 mg daily (12 w)	4 w	35%	−24% (−45% to −6%)
IO Monotherapy Trials									
Gorin et al. (2021) [19]	Pilot	17	cT2a-T4N(any) M0 or T(any) N1M0	100%	94%	Nivolumab 3 mg/kg every 2 w × 3 cycles	Within 7 d	0% (all SD)	SPD: –1.5% (–8.1% to +4.5%)
Carlo et al. (2022) [20]	Pilot	18	^f^ High risk, localized	100%	100%	Nivolumab every 2 w × 4 cycles	Between 7–14 d	0% (all SD)	+0.85% (−6.2% to +7.9%)

BID twice a day; d: day; h: hour, IO: immune checkpoint inhibitor; m: months; ORR: objective response rate; SPD: sum of the product of the two largest perpendicular diameters; tx: treatment; VEGFR-TKI: vascular endothelial growth factor receptor-tyrosine kinase inhibitor; w: week. ^a^ Studies with only metastatic patients and retrospective studies were excluded in this table; ^b^ Of the 12 patients, 9 received sorafenib and 3 received a placebo. ORR and percentage reduction in the primary tumors reported here are the response rates in the sorafenib cohort; ^c^ Mean, not median; ^d^ sunitinib was continued until the tumor became resectable, the patient experienced unacceptable toxicity or disease progression. Metastatic patients with residual disease restarted sunitinib within 8 weeks post-surgery while patients with no residual disease after surgery did not receive further sunitinib therapy. ^e^ Localized with need for the optimal preservation of renal parenchyma based on (1) radical or partial nephrectomy would yield a glomerular filtration rate of less than 30 mL/minute/1.73 m^2^ and/or (2) anticipated increased risk of morbidity with partial nephrectomy due to high complexity (R.E.N.A.L. score 10 to 12) or hilar tumor location; ^f^ high risk is defined as a 12 year probability of metastases of ≥20% as per a preoperative nomogram.

**Table 2:** Ongoing neoadjuvant IO and VEGFR-TKI/IO combination trials.

Trial	Phase	N	Agent	Inclusion Criteria	Primary Outcome	Key Secondary Outcome(s)
Immunotherapy-based combination trials						
NCT05148546 (NESCIO) [27,30]	II	69	Nivolumab, nivolumab + ipilimumab, nivolumab + relatlimab	Clear cell RCC cT1b-cT2aN0M0 and grade 4, cT2bN0M0 and grade 3, cT3-4N0M0 grade(any), cT(any)N1M0 (fully resectable)	Pathologic PR or CR	ORR, RFS, EFS, safety, distant metastases and local recurrence, surgical morbidity, biomarker correlatives
NCT04028245 (SPARC-1) [28,31]	I	14	Canakinumab + Spartalizumab	Clear cell RCC cT1b-T4N(any)M0, T(any)N1M0	Percent of subjects proceeding to RN	ORR, biomarker correlatives
NCT05024318 (NAPSTER) [29,32]	II	20	SABR +/− pembrolizumab	RCC with clear cell, rhabdoid or sarcomatoid components cT1b-T3, N0-N1, M0 or low volume M1 planned for nephrectomy	MPR, biomarker correlatives	Safety, biomarker correlatives
VEGFR-TKI/IO combination trials						
NCT04393350 [33]	II	17	Lenvatinib + pembrolizumab	RCC with clear cell component ≥cT3Nx or T(any)N+ or deemed unresectable, M0	ORR	DFS, OS, Safety
NCT05485896 [34]	II	23	Lenvatinib + pembrolizumab	Clear cell RCC cT(any)N1M(any), cT(any)N(any)M1, cT3-4N(any)M(any), all lesions can be excised or ablated	ORR, safety	PFS, Tumor viability assessment
NCT05319015 [35]	II	30	Lenvatinib + pembrolizumab	RCC, any subtype cT3-4N0-1M0-1 with level 2–4 IVC TT	DCR, local/metastatic progression, 90-day post-op complications	Surgical outcomes, survival outcomes
NCT04995016 (PANDORA) [36]	II	18	Axitinib + pembrolizumab	RCC with clear cell component ≥cT3Nx or T(any)N+ or deemed unresectable by surgeon	MPR	pCR, ORR, DFS, OS, safety
NCT05969496 (NEOPAX) [37,38]	II	17	Axitinib + pembrolizumab	Clear cell RCC cT3b-T4, N0-1, M0-1	Change in IVC TT size/extent	Surgical complications, PFS, OS, safety
NCT05172440 [39,40]	II	20	Axitinib + tislelizumab	Clear cell RCC cT2-T3N0M0	ORR	Surgical outcome, DFS, safety, biomarker correlatives

CR: complete response; DCR: disease control rate; DFS: disease free survival; EFS: event free survival; IO: immune checkpoint inhibitor; IVC TT: inferior vena cava tumor thrombus; MPR: major pathologic response; ORR: objective response rate; OS: overall survival; pCR: pathologic complete response; PFS: progression free survival; PR: partial response; RCC: renal cell carcinoma; RFS: recurrence free survival; RN: radical nephrectomy; SABR: stereotactic ablative radiotherapy; VEGFR-TKI: vascular endothelial growth factor receptor-tyrosine kinase inhibitor.
